# Monitoring of circulating tumour DNA in advanced pancreatic ductal adenocarcinoma predicts clinical outcome and reveals disease progression earlier than radiological imaging

**DOI:** 10.1002/1878-0261.13472

**Published:** 2023-06-28

**Authors:** Karin Hestnes Edland, Kjersti Tjensvoll, Satu Oltedal, Ingvild Dalen, Morten Lapin, Herish Garresori, Nils Glenjen, Bjørnar Gilje, Oddmund Nordgård

**Affiliations:** ^1^ Department of Hematology and Oncology Stavanger University Hospital Norway; ^2^ Section of Biostatistics, Department of Research Stavanger University Hospital Norway; ^3^ Department of Oncology Haukeland University Hospital Bergen Norway; ^4^ Department of Chemistry, Bioscience and Environmental Technology, Faculty of Science and Technology University of Stavanger Norway

**Keywords:** circulating tumour DNA, ctDNA, disease/treatment monitoring, *KRAS*, pancreatic cancer, pancreatic ductal adenocarcinoma

## Abstract

Pancreatic ductal adenocarcinoma (PDAC) is a lethal disease with a need for better tools to guide treatment selection and follow‐up. The aim of this prospective study was to investigate the prognostic value and treatment monitoring potential of longitudinal circulating tumour DNA (ctDNA) measurements in patients with advanced PDAC undergoing palliative chemotherapy. Using *KRAS* peptide nucleic acid clamp‐PCR, we measured ctDNA levels in plasma samples obtained at baseline and every 4 weeks during chemotherapy from 81 patients with locally advanced and metastatic PDAC. Cox proportional hazard regression showed that ctDNA detection at baseline was an independent predictor of progression‐free and overall survival. Joint modelling demonstrated that the dynamic ctDNA level was a strong predictor of time to first disease progression. Longitudinal ctDNA measurements during chemotherapy successfully revealed disease progression in 20 (67%) of 30 patients with ctDNA detected at baseline, with a median lead time of 23 days (*P* = 0.01) over radiological imaging. Here, we confirmed the clinical relevance of ctDNA in advanced PDAC with regard to both the prediction of clinical outcome and disease monitoring during treatment.

## Graphical abstract

The authors collected 425 serial plasma samples from 81 patients with advanced pancreatic ductal adenocarcinoma. The level of circulating tumour DNA (ctDNA) was measured using *KRAS* peptide nucleic acid clamp‐PCR. The results demonstrated that ctDNA measurements at baseline predict clinical outcome and that serial ctDNA measurements can be used for monitoring treatment response.

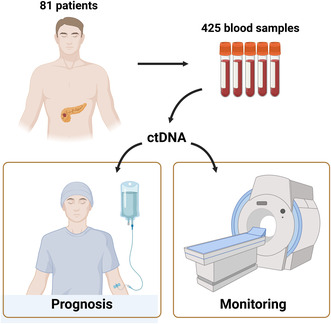

**Abbreviations**
CA19‐9: cancer antigen 19‐9
cfDNA: cell‐free DNA
CHIP: clonal haematopoiesis of indeterminate potential
CI: confidence intervals
Cq: cycle quantification
CT: computed tomography
ctDNA: circulating tumour DNA
ddCq: delta delta cycle quantification
ECOG: Eastern Cooperative Oncology Group
FOLFIRINOX: 5‐fluorouracil, irinotecan and oxaliplatin
HR: hazard ratio
HUH: Haukeland University Hospital
KRAS: Kirsten rat sarcoma virus
nab‐paclitaxel: nanoparticle albumin‐bound paclitaxel
OS: overall survival
PBMC: peripheral blood mononuclear cells
PDAC: pancreatic ductal adenocarcinoma
PFS: progression‐free survival
PNA: peptide nucleic acid
RECIST: response evaluation criteria in solid tumours
SUH: Stavanger University Hospital

## 1 Introduction

Pancreatic ductal adenocarcinoma (PDAC) is a lethal disease with a mortality rate close to the incidence rate and is predicted to be the second leading cause of cancer‐related death in the US in 2030 [1]. Surgery remains the only curative treatment, but more than 80% of patients with PDAC present with unresectable disease [2]. The 5‐year overall survival (OS) in Norway is approximately 14% for all tumour stages combined, and as low as 3% for metastatic disease [3]. Standard palliative chemotherapy for PDAC is combination therapy with 5‐fluorouracil, irinotecan and oxaliplatin (FOLFIRINOX) or nanoparticle albumin‐bound paclitaxel (nab‐paclitaxel) and gemcitabine. Gemcitabine monotherapy is an alternative for patients who, due to performance status and/or comorbidity, cannot receive combination therapy [2].

Computed tomography (CT) complemented by measurement of serum CA19‐9 is routinely used for monitoring of treatment response. Although imaging is considered the gold standard for tumour assessment, the dense fibrotic stroma in PDAC complicates the interpretation of CT‐scans, and challenges exist concerning intra‐ and inter‐observer variability even when using the standardised RECIST criteria [4, 5]. Multiple CT scans are also expensive, require a lot of human resources, and the contrast agents may be nephrotoxic. A problem regarding CA19‐9 is that it may be elevated in patients with PDAC due to other reasons than cancer, such as inflammation and biliary tract obstruction [6]. In addition, Lewis antigen‐negative patients do not express CA19‐9 [7] but seem to have particularly aggressive disease [8].

Circulating tumour DNA (ctDNA) is an emerging tool that may be a useful supplement to traditional biopsies, imaging, and CA19‐9 in the diagnosis, prognostic stratification, and treatment evaluation of PDAC [9, 10]. ctDNA is DNA that is released from apoptotic and necrotic tumour cells into the bloodstream [10]. Several studies, including multiple meta‐analyses, support the prognostic potential of ctDNA detection in plasma, demonstrating that patients in whom ctDNA is detected have a significantly worse prognosis than those in whom it is not detected [11, 12, 13, 14, 15, 16]. Some studies have also explored the relationship between ctDNA and imaging, showing associations between the dynamics of ctDNA and the radiological response [11, 16, 17, 18, 19, 20, 21, 22]. These studies are only observational and evidence of a benefit of ctDNA‐based treatment decisions is still lacking.

More than 90% of PDACs harbour mutations in *KRAS* [23], and there is little discrepancy between *KRAS* mutations found in the tumour and in plasma [21]. Thus, *KRAS* mutations can act as a surrogate for ctDNA detection. Peptide nucleic acid (PNA) clamp real‐time PCR is a highly sensitive method for detecting *KRAS* mutations [24]. Our implementation of the method can detect mutant alleles with a variant allele frequency as low as 0.01% if provided enough input material [24]. Maximising the amount of DNA input is also important for sensitive quantification of ctDNA [10]. Our PNA clamp PCR method allows for up to 200 ng of input DNA (around 60 000 genomes) per reaction, which is more than most other methods [24].

The aim of this prospective study was to evaluate the prognostic potential of detecting ctDNA in plasma from patients with advanced PDAC, as well as exploring ctDNA quantification as a monitoring tool in patients undergoing palliative chemotherapy. We compared ctDNA levels to radiological response evaluations according to the RECIST 1.1 criteria and serum CA19‐9 levels.

## 2 Materials and methods

This manuscript was prepared according to the REMARK guidelines [25].

### 2.1 Population and study design

We prospectively included 81 patients (Table 1) with histologically confirmed advanced PDAC from Stavanger University Hospital (SUH; *n* = 63) and Haukeland University Hospital (HUH; *n* = 18) and collected a total of 425 peripheral blood plasma samples between September 2012 and February 2021. The median patient age was 67 years and 40% were women. All patients were inoperable; 69 had metastatic disease and 12 locally advanced disease. The median follow up‐time was 7.6 months (range 0.3–69.0 months). The patients received chemotherapy according to national Norwegian guidelines at the time of inclusion [26]; 36 patients were treated with FOLFIRINOX, 38 with nab‐paclitaxel/gemcitabine and seven with gemcitabine monotherapy. Treatment was administered until disease progression, unacceptable toxicity, patient's refusal, or patient's need to pause treatment. Patients with a good performance status were offered second‐line treatment after disease progression. For ctDNA analysis, samples of peripheral blood (9 mL in EDTA tubes) were drawn before initiation of chemotherapy and then every 4 weeks thereafter. All samples were processed within 2 h of collection. CT was performed at baseline and every 8 weeks during chemotherapy, or earlier if progression was suspected. Radiology was evaluated according to the respRECIST 1.1 criteria [27]. Serum CA19‐9 levels were monitored at baseline and every 4 weeks. Follow‐up data were collected from hospital records. Plasma samples from 29 volunteers without cancer were analysed and included as a control group. All patients and volunteers provided signed informed consent prior to participation. The project was approved by the Regional Committee for Medical and Health Research Ethics (REK Vest 2011/475, 2013/1743, and 2015/2011). Experimental protocols followed the Declaration of Helsinki.

**Table 1:** Baseline patient characteristics. All data are presented as *n* (%). *P‐*values from Fisher's exact test; bold face *P* values indicate statistically significant differences between patients with ctDNA detected at baseline and those with ctDNA not detected at baseline. Two patients did not have baseline blood samples.

Variable	All patients (*n* = 81)	ctDNA detected (*n* = 44)	ctDNA not detected (*n* = 35)	*P* value
Age^a^				0.373
> 67 years	36 (44)	18 (41)	18 (51)	
≤ 67 years	45 (56)	26 (59)	17 (49)	
Sex				**0.005**
Female	32 (40)	11 (25)	20 (57)	
Male	49 (60)	33 (75)	15 (43)	
Primary tumour location				**0.001**
Head	27 (33)	10 (23)	16 (46)	
Body	15 (19)	9 (20)	5 (14)	
Tail	16 (20)	15 (34)	1 (3)	
Unknown or multiple	23 (28)	10 (23)	13 (37)	
Grade				**0.024**
I	5 (6)	0 (0)	5 (14)	
II	22 (27)	9 (21)	12 (34)	
III	12 (15)	8 (18)	3 (9)	
Unknown	42 (52)	27 (61)	15 (43)	
Clinical T stage				0.428
T1	1 (1)	0 (0)	1 (3)	
T2	16 (20)	10 (23)	6 (17)	
T3	17 (21)	11 (25)	5 (14)	
T4	38 (47)	19 (43)	18 (51)	
Tx	9 (11)	4 (9)	5 (14)	
Clinical N stage				0.065
N0	30 (37)	10 (23)	19 (54)	
N1	35 (43)	21 (48)	13 (37)	
N2	3 (4)	2 (5)	1 (3)	
Nx	13 (16)	11 (25)	2 (6)	
Clinical M stage				**0.004**
M0	12 (15)	2 (5)	10 (29)	
M1	69 (85)	42 (95)	25 (71)	
Metastasis location				**< 0.001**
None	12 (15)	2 (5)	10 (29)	
Other organs than liver	16 (20)	6 (14)	10 (29)	
Liver	53 (65)	36 (82)	15 (43)	
ECOG status				**0.009**
0	18 (22)	4 (9)	13 (37)	
1	48 (60)	29 (66)	18 (51)	
2	15 (18)	11 (25)	4 (11)	
First‐line treatment				0.246
FOLFIRINOX	36 (44)	19 (43)	15 (43)	
Nab‐paclitaxel/gemcitabine	38 (47)	19 (43)	19 (54)	
Gemcitabine	7 (9)	6 (14)	1 (3)	
CA19‐9^a^				0.806
> 626.5 kU·L^−1^	40 (49)	26 (59)	14 (56)	
≤ 626.5 kU·L^−1^	41 (51)	18 (41)	11 (44)	
Study site				0.399
SUH	63 (78)	37 (84)	26 (74)	
HUH	18 (22)	7 (16)	9 (26)	

a Median value used as cut‐off.

### 2.2 Extraction of circulating nucleic acids from plasma

All blood samples were subjected to a Lymphoprep density gradient centrifugation according to the manufacturer's instructions. Peripheral blood mononuclear cells (PBMCs) were lysed in RLT buffer from the AllPrep DNA/RNA/Protein Mini kit (Qiagen, Hilden, Germany). Plasma and PBMC samples were stored at −80 °C until further processing.

Nucleic acid extraction from 1 mL (first five patients) or 4 mL of plasma (diluted 1 : 1 with 0.9% NaCl during density gradient centrifugation) was performed using the QIAamp Circulating Nucleic Acid kit (Qiagen) following the manufacturer's instructions. ctDNA was eluted in 50 μL of Buffer AE. The concentration of cell‐free DNA was measured using a NanoDrop spectrophotometer (median concentration 4 ng·μL^−1^, ranging from below the detection limit to 79 ng·μL^−1^). DNA was extracted from PBMCs using the AllPrep DNA/RNA/Protein Mini kit (Qiagen) according to the manufacturer's protocol. All samples were then stored at −80 °C until further analysis.

### 2.3 Detection of ctDNA by *KRAS* PNA clamp PCR

*KRAS* PNA clamp PCR was performed as described previously [11] using an Mx3005P real‐time PCR instrument (Agilent, Santa Clara, CA, US). Five microlitres or a maximum of 100 ng of plasma cell‐free DNA (cfDNA) was analysed per reaction. All samples were analysed in duplicate and the average value used for calculations. Every sample was analysed with and without the presence of a PNA clamp, which binds preferentially to the wild‐type *KRAS* gene and suppresses its amplification. The PCR amplification with a PNA clamp was compared to the PCR amplification without a PNA clamp to determine the relative level of mutated *KRAS*. This was accomplished by computing ΔCq, which was calculated as ΔCq = Cq_(+PNA)_ – Cq_(−PNA)_. A positive and negative control sample was included on every plate. For the positive control, we used a 1 : 100 dilution of DNA from the LS174T (RRID:CVCL_1384; European collection of cell cultures) colorectal carcinoma cell line (heterozygous GGT > GAT codon 12 *KRAS* mutation [c.35G > A]) in DNA from the Caco‐2 (RRID:CVCL_0025; European collection of cell cultures) colorectal carcinoma cell line (*KRAS* wt). For the negative control, we used cfDNA from a healthy individual. To define a cut‐off for plasma *KRAS* mutation status, we analysed plasma samples from 29 healthy individuals. The lowest ΔCq was 11.27 (defined as ΔCq_wt,min_). To enhance the interpretability of our *KRAS* mutation measurements, we computed ΔΔCq values, which were calculated as ΔΔCq = ΔCq_wt,min_ − ΔCq_sample_. Samples with ΔΔCq > 0 were considered positive for *KRAS*‐mutated ctDNA, and ΔΔCq values < 0 were set to 0 in the statistical analyses. If a sample had only one replicate with amplification when the PNA clamp was present, this replicate was used in further calculations. If a sample had no amplification signal in both replicates with a PNA clamp, ctDNA was considered as being not detected. For patients with *KRAS* mutations detected in any plasma sample, DNA from PBMCs was analysed to ensure that the mutations were not due to clonal haematopoiesis of indeterminate potential (CHIP). Cell lines have been authenticated in the last 3 years using short tandem repeat profiling. All cell‐line experiments were performed with mycoplasma‐free cells.

### 2.4 Statistical analysis

Primary endpoints were progression‐free survival (PFS) and OS. PFS was defined as the time from signed consent until first radiological progression according to the RECIST 1.1 criteria [27] or death due to any cause. OS was defined as the time from signed consent until death from any cause. Patients still alive at the time of analysis were censored at the last follow‐up date. All patients had progressed at the time of statistical analysis. PFS and OS were estimated using the Kaplan–Meier method, and the log rank test was used to compare survival curves. To avoid immortal time bias, time to progression for samples taken 4 weeks after initiation of chemotherapy was calculated from the date of sampling. Univariable and multivariable Cox proportional hazards regression models were used to estimate hazard ratios, associated 95% confidence intervals (CIs) and *P‐*values for potential prognostic factors (listed in Table 2). Due to several cases of missing values for T‐ and N‐stage and tumour grade, these variables were not included in the multivariable modelling. The initial multivariable Cox models included ctDNA detected at baseline (yes/no), age, sex, ECOG performance status (0/1/2), first treatment (FOLFIRINOX, nab‐paclitaxel/gemcitabine, gemcitabine monotherapy), metastasis status and study site (SUH/HUH) as explanatory variables. Backward elimination of variables according to the likelihood ratio statistic was used in the multivariable Cox regressions, successively removing variables with a *P*‐value > 0.1. The proportional hazards assumption was tested with log minus log‐plots and Schoenfeld‐residuals.

**Table 2:** Univariable Cox regression. Significant *P* values are in bold.

Parameter	*n*	Progression‐free survival		Overall survival	
		Hazard ratio (95% CI)	*P* values	Hazard ratio (95% CI)	*P* values
Baseline ctDNA status (detected vs. not detected)	79	2.52 (1.53–4.14)	**< 0.001**	2.13 (1.32–3.44)	**0.002**
Baseline ctDNA level (per unit))	79	1.10 (1.06–1.15)	**< 0.001**	1.10 (1.05–1.15)	**< 0.001**
1‐month ctDNA status	58	2.95 (1.66–5.26)	**< 0.001**	2.91 (1.61–5.26)	**< 0.001**
1‐month ctDNA level	58	1.11 (1.05–1.16)	**< 0.001**	1.12 (1.06–1.18)	**< 0.001**
Age (per year)	80	1.00 (0.98–1.02)	0.88	0.99 (0.97–1.02)	0.50
Sex (male vs. female)	80	1.43 (0.90–2.28)	0.13	1.47 (0.90–2.41)	0.13
ECOG status	80		**< 0.001**		**< 0.001**
0		Reference		Reference	
1		0.91 (0.53–1.59)	0.75	1.01 (0.58–1.77)	0.97
2		3.56 (1.66–7.63)	**0.001**	6.16 (2.81–13.48)	**< 0.001**
First‐line treatment	80		**< 0.001**		**< 0.001**
FOLFIRINOX		Reference		Reference	
Nab‐paclitaxel/gemcitabine		1.56 (0.98–2.49)	0.061	1.71 (1.05–2.78)	0.032
Gemcitabine		7.19 (2.78–18.59)	**< 0.001**	9.52 (3.50–25.89)	**< 0.001**
Study site (SUH vs. HUH)	80	1.88 (1.07–3.31)	**0.029**	1.98 (1.10–3.59)	**0.023**
Tumour location (head vs. body/tail)	58	0.58 (0.35–1.00)	0.051	0.76 (0.45–1.30)	0.32
Metastasis location	80		**0.022**		**0.048**
None		Reference		Reference	
Other organs than liver		1.30 (0.60–2.82)	0.50	0.98 (0.44–2.19)	0.95
Liver		2.28 (1.16–4.47)	**0.016**	1.88 (0.96–3.68)	0.065

Dynamic prediction of progression based on longitudinal measures of ctDNA levels were assessed using joint models in r (version 4.1.2). Due to the endogenous nature of ctDNA levels, standard survival analysis with time‐varying covariates may give biased results [28]. First, a mixed linear model was fitted to the longitudinal ctDNA levels, in which the fixed effect of time was modelled using natural cubic splines (ncs) with three knots, and applying random intercept and random effect of time (ncs with two knots). Time since last infusion (1–2, 3–7, and > 7 days) was added as a fixed factor. Observations after the time of first progression were excluded from the analysis. Cox regression was used to model time to first progression, applying baseline predictors site, age, sex, ECOG performance status, treatment regime at start of treatment and metastatic status (no metastases, metastases but not in liver and metastases in liver). The joint model was derived with these two models as input using function jm in package jmbayes2, in which the estimated current value of the ctDNA level was linked to time to first progression. Other functional forms of the association were explored (i.e. letting time to progression depend on the slope of the estimated trajectory of ctDNA or on the scaled area under the trajectory). The estimated effect of ctDNA level on time to first progression is presented as a hazard ratio (HR) with accompanying 95% CI. Model predictions for a selection of patients representing typical longitudinal trajectories of ctDNA are presented in plots.

Similar joint modelling was performed for CA19‐9 levels as a predictor of time to first progression. Due to an extremely skewed distribution, we used log2‐transformed CA19‐9 levels in the longitudinal model, and did not adjust for time since the last infusion. Supplementary analyses were done excluding the HUH patients.

ctDNA levels at baseline, after 1 and 2 months of therapy, and at time of progression were compared using the Mann–Whitney *U*‐test. Lead times for detection of disease progression were compared to zero time difference using Wilcoxon signed rank test with continuity correction. Patient characteristics were presented as counts and percentages and compared between groups using Fisher's exact test. *P*‐values ≤ 0.05 were considered significant. All statistical analyses were carried out using spss version 26.0 (IBM, New York, US)and r version 4.1.2 and 4.2.1.

## 3 Results

### 3.1 Patient characteristics and ctDNA at baseline

**Fig. 1:**
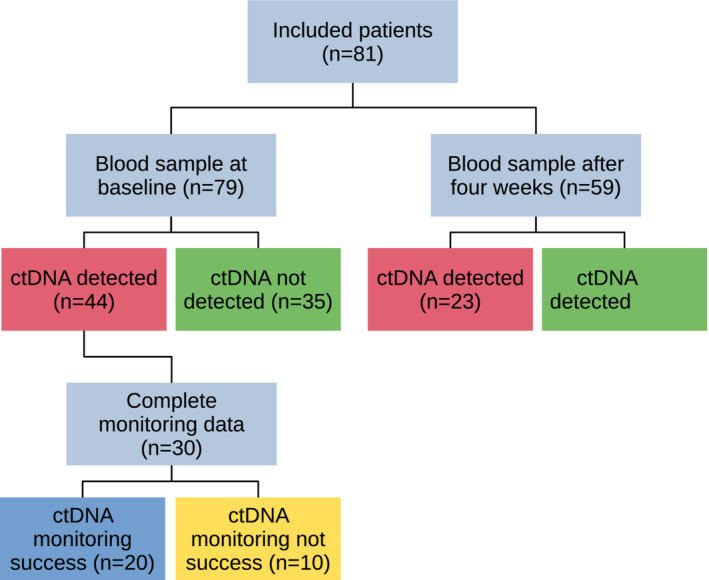
Patient flow diagram showing the number of patients included and the main results of ctDNA measurements in the various parts of the study.

We measured ctDNA levels in 81 patients with advanced PDAC (Fig. 1) before and during chemotherapy, using mutations in the *KRAS* gene (codon 12/13) as ctDNA marker. Baseline patient characteristics are summarised in Table 1. Of the 81 patients, 79 had baseline plasma samples available. *KRAS* mutations were detected in 44 of 79 (56%) plasma samples at baseline, in 42 of 62 (63%) samples from patients with metastatic disease, and in two of 12 (17%) samples from patients with locally advanced disease (*P* = 0.004). Sex, primary tumour location and differentiation grade, clinical M stage, liver metastases, and ECOG status were significantly associated with baseline ctDNA detection (Table 1). There were no significant differences in ctDNA status between the groups receiving different first‐line treatment, having different clinical T and N stage, between the patients with baseline serum CA19‐9 levels above or below the median, or between the two study sites.

### 3.2 Prognostic value of ctDNA

**Fig. 2:**
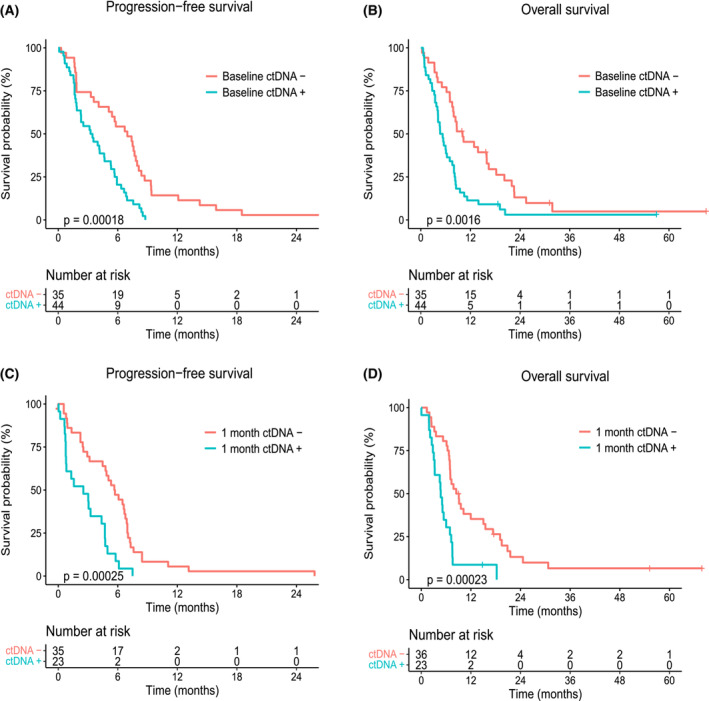
Kaplan–Meier survival estimates according to ctDNA detection. (A) Progression‐free survival (PFS) according to baseline ctDNA detection (Baseline ctDNA + or −). (B) Overall survival (OS) according to baseline ctDNA detection (Baseline ctDNA + or −). (C) PFS according to ctDNA detection after 1 month of chemotherapy (1 month ctDNA + or −). (D) OS according to ctDNA detection after 1 month of chemotherapy (1 month ctDNA + or −). One patient experienced disease progression before the second sample collection, and was therefore only included in the analysis of OS (D). Censored patients are indicated by plus symbols (+) on the survival estimate curves. *P*‐values correspond to log‐rank tests for survival differences.

At the time of analysis, all patients had experienced disease progression and 75 of 81 (93%) patients had died. Kaplan–Meier analyses of ctDNA detected versus not detected at baseline demonstrated a median PFS of 3.3 (95% CI 1.4–5.1) months versus 7.0 (4.9–9.1) months (*P* < 0.001; Fig. 2A) and a median OS of 4.7 (3.2–6.2) months versus 10.1 (5.0–15.1) months (*P* = 0.002; Fig. 2B). Analysis of ctDNA status 1 month after initiation of chemotherapy revealed a median PFS of 2.5 (0.0–5.2) months versus 5.7 (4.1–7.4) months (*P* < 0.001; Fig. 2C) and a median OS of 4.7 (3.8–5.6) months versus 8.4 (5.8–11.1) months (*P* < 0.001; Fig. 2D) for patients with ctDNA detected versus not detected, respectively.

In univariable Cox regression, both ctDNA level and detection (yes/no) at baseline and after 1 month of chemotherapy were prognostic factors for PFS and OS (Table 2). ECOG performance status, first‐line treatment, study site and metastatic disease in the liver were also significant predictors. Study site was also significantly associated with baseline ECOG performance status and first‐line treatment (*P* = 0.03 and *P* < 0.001, respectively). In a multivariable model (Table 3), ctDNA detection at baseline was an independent prognostic factor for PFS (HR 2.1, 95% CI 1.2–3.8, *P =* 0.010) and OS (HR 2.0, 95% CI 1.2–3.5, *P =* 0.010). Other independent predictors of PFS and OS were age, ECOG performance status, and first‐line treatment. Furthermore, liver metastasis was an independent prognostic factor for PFS.

**Table 3:** Multivariable Cox regression. Significant *P* values are in bold. Based on 79 patients.

Parameter	Progression‐free survival		Overall survival	
	Hazard ratio (95% CI)	*P* value	Hazard ratio (95% CI)	*P* value
Baseline ctDNA status (detected vs. not detected)	2.14 (1.20–3.84)	**0.010**	2.04 (1.19–3.52)	**0.010**
Age (per year)	0.97 (0.94–1.00)	**0.030**	0.96 (0.93–0.99)	**0.004**
ECOG status		**< 0.001**		**< 0.001**
0	Reference		Reference	
1	0.53 (0.26–1.08)	0.081	0.56 (0.28–1.09)	0.089
2	2.45 (0.97–6.18)	0.057	4.83 (1.89–12.37)	**0.001**
First‐line treatment		**< 0.001**		**< 0.001**
FOLFIRINOX	Reference		Reference	
Nab‐paclitaxel/gemcitabine	2.44 (1.32–4.49)	**0.004**	2.35 (1.27–4.36)	**0.006**
Gemcitabine	6.67 (2.46–18.09)	**< 0.001**	8.88 (3.12–25.30)	**< 0.001**
Metastasis location		0.098		
None	Reference			
Other organs than liver	1.55 (0.67–3.59)	0.303		
Liver	2.25 (1.04–4.87)	**0.038**		

### 3.3 Joint modelling and dynamic predictions of time to progression

**Fig. 3:**
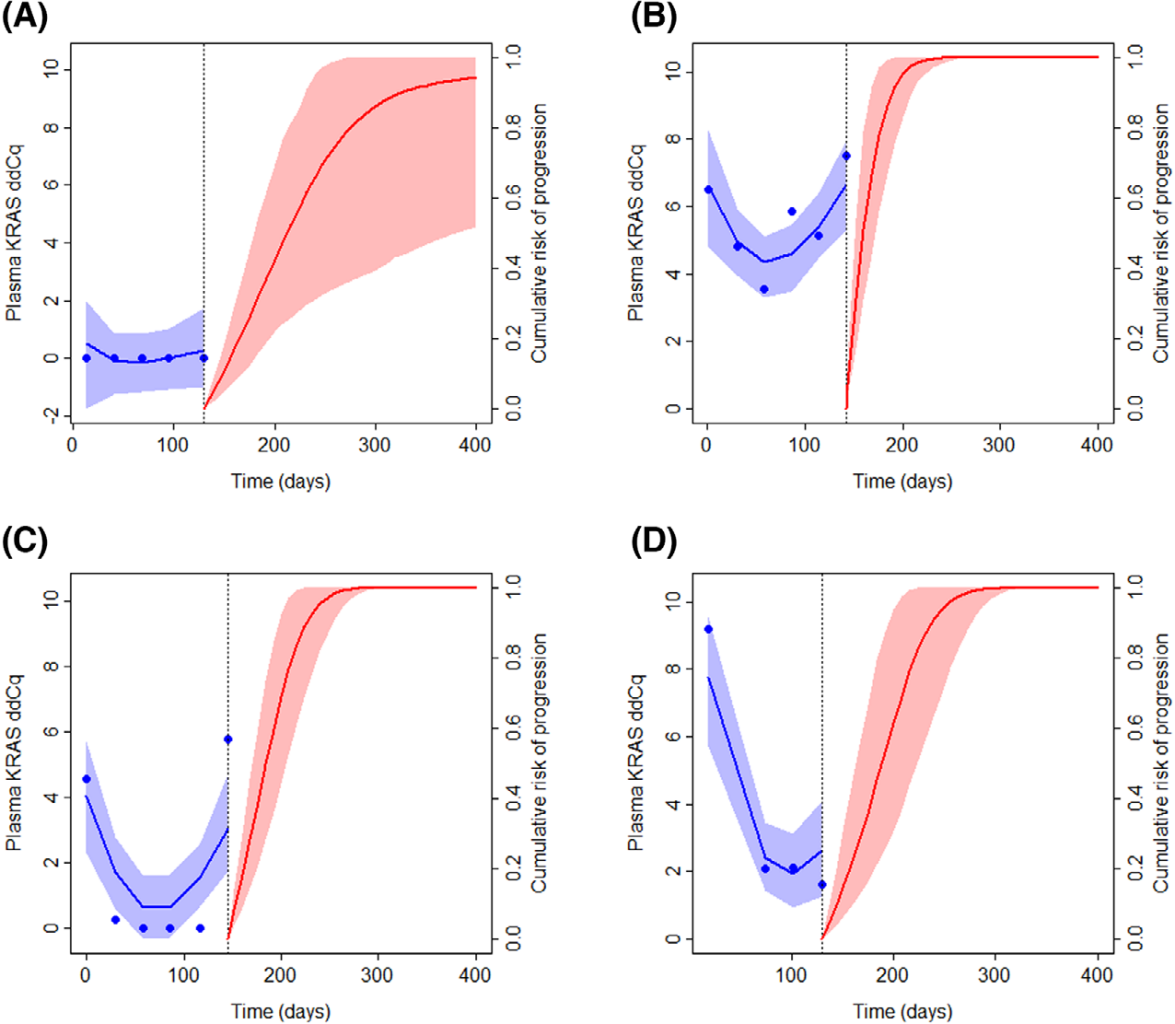
Dynamic prediction of first progression for select patients. The left‐hand side of the plots shows the observed and fitted longitudinal trajectory of ctDNA level (plasma KRAS ddCq) from baseline to approximately 140 days; the right‐hand side shows the predicted cumulative risk of progression from the time of the last included plasma KRAS ddCq observation. (A) 72‐year‐old woman from Stavanger University Hospital (SUH) with liver metastases, Eastern Cooperative Oncology Group performance status (ECOG PS) = 0, first‐line medication FOLFIRINOX, time of first progression 284 days. (B) 51‐year‐old woman from SUH with liver metastases, ECOG PS = 1, first‐line medication nab‐paclitaxel/gemcitabine, time of first progression 162 days. (C) 72‐year‐old woman from SUH with liver metastases, ECOG PS = 1, first‐line medication nab‐paclitaxel/gemcitabine, time of first progression 173 days. (D) 52‐year‐old man from Haukeland University Hospital (HUH) with liver metastases, ECOG PS = 1, first‐line medication FOLFIRINOX, time of first progression 249 days. Animations of the developing dynamic predictions over time are shown in Animations [Link], [Link] (corresponding to panels A–D).

We measured ctDNA levels in blood samples obtained monthly during chemotherapy from the included patients. Eighty‐one patients had at least one ctDNA measurement and were included in the joint model, with a total of 298 observations. The complete joint model is presented in Table 4. The effect of ctDNA on time to first progression was highly significant, with an HR of 1.21 (95% CI 1.09–1.34; *P* < 0.001). Similarly, 77 patients had at least one CA19‐9 measurement (291 observations total), and for these the HR of log2 CA19‐9 levels with regard to time to first progression was estimated to be 1.02 (0.95–1.10, *P* = 0.61; Table S1). The HR associated with a difference in the slopes of ctDNA levels was not statistically significant (1.08 per 0.01 units difference, 1.00–1.19; *P* = 0.067). The HR for the area under the trajectory of ctDNA levels was similar to the HR for the current value (1.25, 1.09–1.42; *P* < 0.001). For log2 transformed CA19‐9, the HR for slope (per 0.01 units) was estimated to be 1.33 (0.93–1.97, *P* = 0.13) and for area 1.02 (0.94–1.10; *P* = 0.64). Prediction plots for time to first progression based on the joint model in Table 4 are presented for select patients in Fig. 3 and Animations [Link], [Link].

**Table 4:** Joint modelling of longitudinal ctDNA level and time to first progression. Based on 81 patients with a total of 298 ctDNA observations. Statistically significant *P* values are in bold.

Survival submodel	HR (95% CI)	*P* value
Study site (SUH vs. HUH)	1.36 (0.54–3.42)	0.51
Sex (male vs. female)	1.43 (0.84–2.46)	0.19
Age (per year)	0.97 (0.95–1.00)	0.081
ECOG status		
0	Reference	
1	0.53 (0.27–1.03)	0.061
2	2.05 (0.86–4.85)	0.11
Treatment		
FOLFIRINOX	Reference	
Nab‐paclitaxel/gemcitabine	2.20 (1.08–4.76)	**0.026**
Gemcitabine	4.57 (1.62–12.7)	**0.004**
Metastasis location		
None	Reference	
Other organs than liver	1.80 (0.71–4.62)	0.21
Liver	2.23 (1.02–5.26)	**0.046**
ctDNA level (per unit)	1.21 (1.09–1.34)	**< 0.001**

Longitudinal submodel	β (95% CI)	*P* value
Time since baseline		
Spline 1	−1.55 (−2.85, −0.19)	**0.025**
Spline 2	0.38 (−1.56, 2.28)	0.72
Spline 3	7.18 (3.69, 10.5)	**< 0.001**
Time since infusion		
> 7 days	Reference	
3–7 days	−0.01 (−0.95, 0.94)	0.44
1–2 days	0.63 (−0.99, 2.28)	0.99

Excluding the smallest site (HUH), the corresponding HR for ctDNA level was 1.23 (1.11–1.37; *P* < 0.001; based on 63 patients, 245 observations). For the slope of log2 CA‐19‐9 levels (per 0.01 units) it was 1.24 (1.01–1.61, *P* = 0.041; 60 patients, 247 observations).

### 3.4 Disease monitoring by ctDNA

**Fig. 4:**
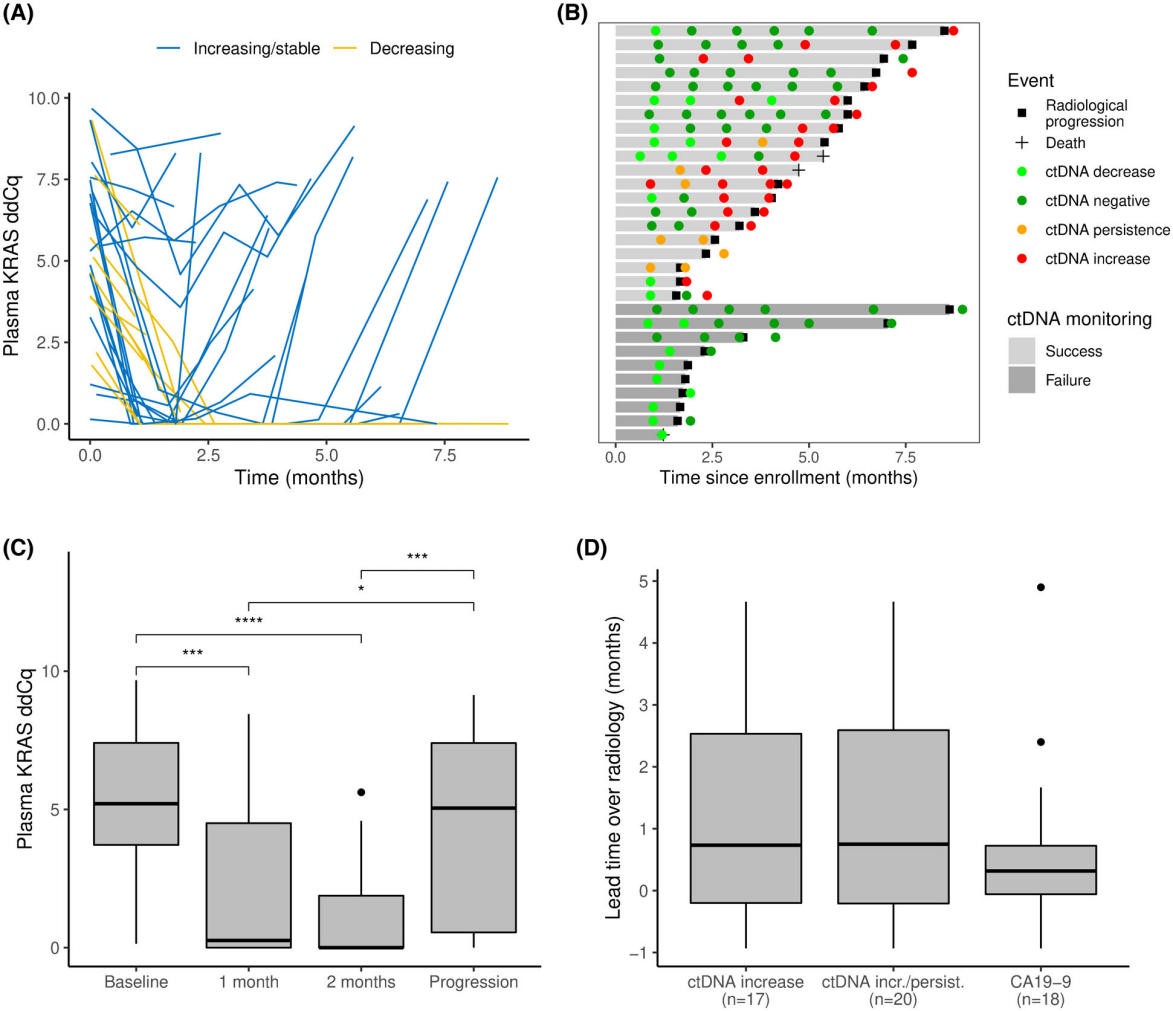
Serial ctDNA measurements during chemotherapy. **(**A) Plasma ctDNA levels (measured by *KRAS* ddCq values), monitored during chemotherapy and up to 1 month after radiological evidence of disease progression. Patients with increasing or stably high ctDNA levels at or before the time of progression are shown as blue curves. Patients with ctDNA levels that only decrease are shown as yellow curves. (B) Swimmerplot showing ctDNA monitoring of the 30 patients included in the monitoring analysis. Patient timelines are shown horizontally, with grey bars indicating the time until radiologically confirmed disease progression (black square) or death (black cross), whichever came first. Light grey bars show patients with persistent or increasing ctDNA levels before or until 1 month after radiological evidence of progression (Success), and dark grey bars show patients without such an observation (Failure). ctDNA measurements after initiation of chemotherapy and up to 1 month after radiological progression are shown as circles, coloured according to the level of ctDNA (light green: decrease, dark green: negative, orange: persistence, red: increase). An increase was defined as at least a doubling (Cq value increased by at least 1) or as any increase in cases with a preceding negative sample; decrease was defined as a more than 50% reduction in ctDNA level compared to the previous sample (Cq reduction > 1) or as any reduction below ddCq = 1. ctDNA levels between these limits were categorised as persistence. (C) Boxplot showing plasma KRAS ddCq values at baseline, after 1 and 2 months of chemotherapy, and at the time of progression. Significant pairwise comparisons (Wilcoxon rank‐sum test) are indicated by brackets: *P < 0.05, ***P < 0.001, ****P < 0.0001. The upper whisker extends from the box to the largest value no further than 1.5 times the interquartile range from the upper box border. (D) Lead time of ctDNA and CA19‐9 monitoring over radiological monitoring. Only patients for which the marker indicated disease progression are included and their numbers are shown in parentheses below the *x*‐axis. The upper whisker extends from the box to the largest value no further than 1.5 times the interquartile range from the upper box border.

To be able to compare ctDNA dynamics and radiological monitoring, we restricted our subsequent analyses to the 30 patients with *KRAS* mutations in the baseline blood sample who had at least two blood samples collected, including at least one blood sample collected less than 1 month before or after the date of radiologically confirmed disease progression. The majority of patients (*n* = 20) had either persistently high ctDNA levels (*n* = 3) or initially decreasing ctDNA levels that increased again at the time of or earlier than radiological disease progression (*n* = 17), whereas 10 patients had decreasing ctDNA levels that did not reflect disease progression (Fig. 4A,B). The ctDNA levels were significantly lower after 1 and 2 months of chemotherapy than at baseline (both *P* < 0.001; Fig. 4C) and at the time of radiological disease progression (*P* = 0.01 and *P* < 0.001, respectively). The median lead time of ctDNA‐based detection of disease progression over radiological detection was 23 days (*P* = 0.01; Fig. 4D). When we used ctDNA increase only as a requirement for detection of disease progression, and not ctDNA persistence, the median lead time over radiology was 22 days (*P* = 0.02). We performed a similar analysis of CA19‐9 and found a median lead time of 9.5 days (*P* = 0.03) for the 18 (of 30, 60%) patients who had CA19‐9 increase (> 50%) at or before the time of radiological evidence of progression (Fig. 4D). For 6 (of 20, 30%) patients who had ctDNA levels reflecting disease progression, there was no CA19‐9 increase observed during therapy. Conversely, ctDNA failed to reflect disease progression for four of 18 (22%) patients with successful CA19‐9 monitoring. Either ctDNA or CA19‐9 revealed disease progression in 24 of 30 (80%) patients. Example plots showing ctDNA and CA19‐9 levels in comparison for selected patients are shown in Fig. S1.

## 4 Discussion

The clinical relevance of liquid biopsies in pancreatic cancer have been investigated in several studies, especially assessment of peripheral blood‐based entities like circulating tumour cells, vesicles and nucleic acids [12, 13, 29]. In the current study we demonstrated the clinical relevance of ctDNA in advanced PDAC with regard to both predicting outcome and disease monitoring during chemotherapy. Both the ctDNA level (baseline and after 1 month of therapy) and its dynamic changes during the initial therapeutic phase predicted clinical outcome. Moreover, serial monitoring of ctDNA revealed disease progression at the same time or earlier than radiological imaging in a subgroup of the patients.

In general, ctDNA detection at baseline was associated with increased disease burden in our study (Table 1), which is a well‐established connection in many cancers [10]. Interestingly, patients with primary tumours located in the tail and body of the pancreas were more frequently ctDNA‐positive at baseline than patients with tumours in the pancreas head, an observation that seems to be unique. Other studies of ctDNA in PDAC have not observed such an association [12, 30], have only observed a tendency [16], or have not reported such data. Our observation may be related to the notion that tail tumours are associated with more advanced disease and a worse prognosis [31, 32].

Despite approximately 90% of PDACs having mutations in *KRAS* [23, 33], we observed ctDNA with *KRAS* mutations in 56% of the baseline blood samples. This number is similar to observations in other studies of *KRAS‐*mutated ctDNA in advanced PDAC [12, 20, 30]. Although sufficient for prognostic stratification, the number is low with regard to a wide utility in disease monitoring. Therefore, we asked whether the relatively low number of ctDNA‐positive patients in our and similar studies was due to technical or biological factors. Our PNA clamp PCR assay only detects mutations in codons 12 and 13 of *KRAS*, but these variants constitute more than 95% of the reported *KRAS* mutations in PDAC [33]. We also analysed most of the samples in the current study using a sequencing‐based approach and found that 95% of the mutations in *KRAS* occur in codons 12 and 13 [34]. Overall, we observed 89% concordance between the two methods for mutations in codon 12 of 13 ([34] and results not shown), emphasising the robustness of the PNA clamp method. Moreover, the amount and quality of cfDNA input influences ctDNA detection [10]. In the current study we analysed amounts of cfDNA in the range of < 10 to 100 ng per reaction, meaning that the analytical sensitivity should vary between samples. Accordingly, we observed a higher level of ctDNA‐positivity among samples with a higher than median amount of amplifiable input DNA (*P* = 0.007), emphasising the importance of the amount and quality of input cfDNA. Biological aspects also contribute to a low detection rate, as PDAC seems to generally shed less ctDNA than other cancer types [35, 36]. Higher detection rates can be achieved by analysing larger plasma volumes and using markers that are more frequent in ctDNA than point mutations, such as hypermethylation and DNA fragmentation profiles [10, 37, 38, 39, 40].

We demonstrated an independent prognostic value of baseline ctDNA detection in our study, an observation that confirms previously published results, including several meta‐analyses [13, 14]. Our HRs of 2.1 and 2.0 for PFS and OS, respectively, were slightly lower than in our previously published pilot study and the pooled data analyses [11]. This can be due to the new multi‐drug treatments that have become available during our study period, which may have prolonged the survival of patients with high levels of ctDNA. Although ctDNA levels generally decreased during the first weeks of treatment (Fig. 4C), patients with ctDNA detected after 4 weeks of chemotherapy also had shorter PFS and OS than those who did not have any ctDNA detected. The HRs for ctDNA detection at 1 month were even higher than before chemotherapy (2.9 for PFS and OS), suggesting that ctDNA persistence during initial chemotherapy may be a stronger indicator of poor prognosis than baseline detection. To further explore the potential of ctDNA dynamics during first‐line treatment, we performed joint modelling of PFS as a function of the time‐dependent level of ctDNA and found a significant association (HR = 1.21). Correspondingly, we found statistically significant associations with the area under the trajectory but not with the slope of the KRAS trajectory. This model could also be used for subject‐specific dynamic predictions of PFS, which may enhance therapeutic choices in the future [41].

We observed somewhat shorter progression‐free and overall survival among the patients recruited at the main study site (SUH) compared to the secondary site (HUH). This seemed to be related to the lower baseline ECOG performance and the more frequent treatment with the FOLFIRINOX combination at the HUH site. The latter could partly be because the recruitment of patients started later at the secondary site, when FOLFIRINOX was recommended. With regard to the validity of our conclusions, the number of patients with ctDNA detected at baseline was not significantly different between the sites and the prognostic relevance of ctDNA was verified also when excluding the samples from HUH.

Longitudinal analysis of ctDNA levels demonstrated that ctDNA monitoring revealed disease progressions at the same time or earlier than radiological monitoring in 20 of 30 (67%) patients with detectable ctDNA levels at baseline (Fig. 4), with a median lead time of 23 days. These findings were similar to previously published results [20, 22]. For this patient subgroup with detectable levels of ctDNA, ctDNA monitoring may represent a new diagnostic tool to guide treatment changes and avoid futile chemotherapy and associated side effects. On the other hand, the relatively high number of patients without ctDNA detected at baseline and no ctDNA increase at the time of progression weakens the utility of ctDNA as a general monitoring tool in the current clinical setting. We observed the same when using an eight‐gene sequencing‐based approach for ctDNA detection, suggesting that we face a biomedical challenge rather than a technical one [34]. However, ctDNA may have a specific utility in the subset of patients in whom CA19‐9 is not useful for monitoring, as emphasised by our identification of 6/39 (20%) patients with progression indicated by ctDNA but not CA19‐9. ctDNA analysis may also have extended relevance if the future brings targeted therapies to the pancreatic cancer field, as ctDNA has entered the clinic for such applications in other cancer types [42]; ctDNA assessment for tumour genotyping and monitoring of resistance mutations in advanced cancers was recently been recommended by the European Society for Medical Oncology [10].

## 5 Conclusions

In the current study, we confirmed the clinical relevance of ctDNA in advanced PDAC with regard to prediction of outcome and disease monitoring during chemotherapy. To establish the clinical utility of ctDNA in this context, there is a need for prospective interventional studies exploring the survival and quality of life benefits of treatment choices based on ctDNA measurements. Apparently, lower levels of ctDNA in some patients may reduce its utility in monitoring PDAC, but this may be compensated for by applying multiple ctDNA marker types and larger sample volumes. ctDNA monitoring may also be more useful in specific clinical contexts, especially with regard to potential future biologically targeted therapies.

## Conflict of interest

The authors declare no conflict of interest.

## Author contributions

KHE collected clinical data, performed laboratory and statistical analyses, and drafted the manuscript. KT participated in the study design, laboratory analyses, data interpretation and manuscript preparation. ML participated in data analysis, interpretation and manuscript preparation. SO performed laboratory analyses, data interpretation and reviewed the manuscript. ID supervised and performed statistical analyses and prepared the manuscript. HG and NG contributed to patient recruitment, data interpretation and manuscript preparation. BG contributed to the study design and coordination, data interpretation and manuscript preparation. ON contributed to the study design and coordination, statistical analyses, data interpretation, and manuscript preparation. All authors read and approved the final version of the manuscript.

### Peer Review

The peer review history for this article is available at https://www.webofscience.com/api/gateway/wos/peer-review/10.1002/1878-0261.13472.

## Supporting information


**Animation S1.** Dynamic prediction of first progression for a 72‐year‐old woman from Stavanger University SUH with liver metastases, ECOG = 0, first‐line medication FOLFIRINOX, time of first progression 284 days.

**Animation S2.** Dynamic prediction of first progression for a 51‐year‐old woman from SUH with liver metastases, ECOG = 1, first‐line medication nab‐paclitaxel/gemcitabine, time of first progression 162 days.

**Animation S3.** Dynamic prediction of first progression for a 72‐year‐old woman from SUH with liver metastases, ECOG = 1, first‐line medication nab‐paclitaxel/gemcitabine, time of first progression 173 days.

**Animation S4.** Dynamic prediction of first progression for a 52‐year‐old man from HUH with liver metastases, ECOG = 1, first‐line medication FOLFIRINOX, time of first progression.

**Fig. S1.** Plasma ctDNA, serum CA19‐9 levels and radiological monitoring data for selected patients.

**Table S1.** Joint modelling of longitudinal CA19‐9 and time to first progression.

## Data Availability

Data available on reasonable request due to privacy/ethical restrictions.
