# Supplementary figures and images for: Monitoring of circulating tumour DNA in advanced pancreatic ductal adenocarcinoma predicts clinical outcome and reveals disease progression earlier than radiological imaging
Source: Mol Oncol. 2023 Jun 28;17(9):1857–70. doi: 10.1002/1878-0261.13472 (PMC10483602; doi:10.1002/1878-0261.13472)

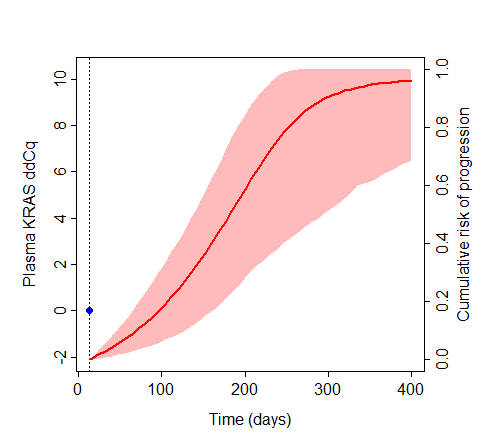

Supplement: Supplementary file 1 — Animation S1. Dynamic prediction of first progression for a 72‐year‐old woman from Stavanger University SUH with liver metastases, ECOG = 0, first‐line medication FOLFIRINOX, time of first progression 284 days. [file MOL2-17-1857-s004.gif]

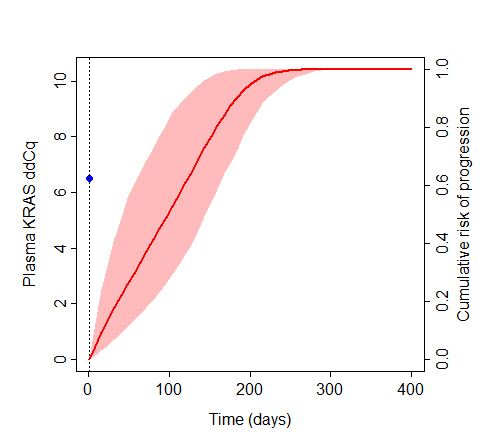

Supplement: Supplementary file 2 — Animation S2. Dynamic prediction of first progression for a 51‐year‐old woman from SUH with liver metastases, ECOG = 1, first‐line medication nab‐paclitaxel/gemcitabine, time of first progression 162 days. [file MOL2-17-1857-s003.gif]

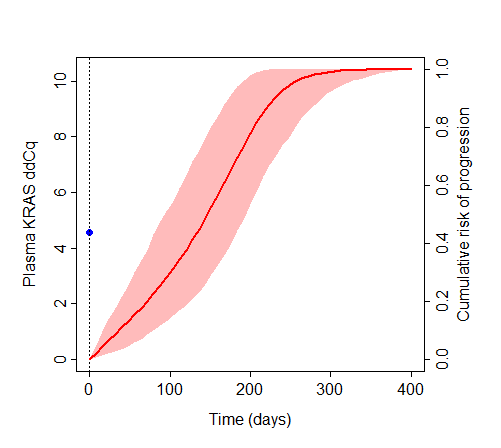

Supplement: Supplementary file 3 — Animation S3. Dynamic prediction of first progression for a 72‐year‐old woman from SUH with liver metastases, ECOG = 1, first‐line medication nab‐paclitaxel/gemcitabine, time of first progression 173 days. [file MOL2-17-1857-s006.gif]

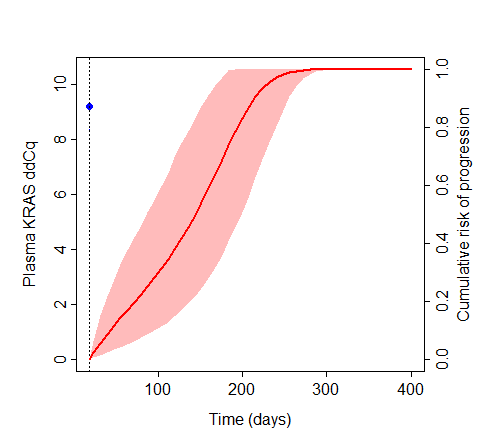

Supplement: Supplementary file 4 — Animation S4. Dynamic prediction of first progression for a 52‐year‐old man from HUH with liver metastases, ECOG = 1, first‐line medication FOLFIRINOX, time of first progression. [file MOL2-17-1857-s001.gif]
