# Supplementary material for: Monitoring of circulating tumour DNA in advanced pancreatic ductal adenocarcinoma predicts clinical outcome and reveals disease progression earlier than radiological imaging
Source: Mol Oncol. 2023 Jun 28;17(9):1857–70. doi: 10.1002/1878-0261.13472 (PMC10483602; doi:10.1002/1878-0261.13472)
Supplement: Supplementary file 2 [file MOL2-17-1857-s002.odt]

Supplemental Table S1: Joint modeling of longitudinal CA19-9 and time to first progression 

| Survival submodel | HR (95% CI) | P value |
| --- | --- | --- |
| Study site (SUH vs HUH) | 1.49 (0.61-3.82) | 0.40 |
| Sex (male vs female) | 1.52 (0.92-2.61) | 0.11 |
| Age (per year) | 0.96 (0.94-0.99) | 0.009 |
| ECOG performance status |  |  |
| 0 | Reference |  |
| 1 | 0.61 (0.32-1.21) | 0.15 |
| 2 | 2.83 (1.22-7.02) | 0.017 |
| Treatment |  |  |
| FOLFIRINOX | Reference |  |
| Nab-paclitaxel/gemcitabine | 2.03 (1.03-4.26) | 0.044 |
| Gemcitabine | 4.62 (1.57-12.9) | 0.009 |
| Metastasis location |  |  |
| None | Reference |  |
| Other organs than liver | 1.72 (0.77-4.01) | 0.20 |
| Liver | 2.69 (1.34-5.75) | 0.003 |
| CA19-9 level (per unit) | 1.02 (0.95-1.10) | 0.61 |
| Longitudinal submodel | β (95% CI) | P value |
| Spline 1 | -3.56 (-6.18, -0.76) | <0.001 |
| Spline 2 | -1.66 (-4.94, 1.28) | 0.29 |
| Spline 3 | 3.14 (-2.70, 8.13) | 0.29 |

CA19-9 levels were log2 transformed. Based on 77 patients with a total of 291 observations of CA19-9. Statistically significant P-values are in bold
